# Lean Mass Index Adjustment of eGFR Strengthens Associations with Cardiometabolic Risk Factors

**DOI:** 10.3390/diagnostics16213540

**Published:** 2026-10-28

**Authors:** Oliver Helk, Charmaine J. M. Lim, Matthäus Metz, Emiel F. M. Wouters, Robab Breyer-Kohansal, Marie-Kathrin Breyer

**Affiliations:** 1 Division for Nephrology and Dialysis, Department of Medicine III, Medical University of Vienna, General Hospital of Vienna, 1090 Vienna, Austria; matthaeus.metz@meduniwien.ac.at; 2 Austrian Academic Institute for Clinical Nutrition, 1090 Vienna, Austria; 3 Ludwig Boltzmann Institute for Lung Health, 1140 Vienna, Austria; charmaine.helk-lim@leadstudy.at (C.J.M.L.); woutersemiel@gmail.com (E.F.M.W.); robab.breyer-kohansal@copd.lbg.ac.at (R.B.-K.); marie-kathrin.breyer@lbg.ac.at (M.-K.B.); 4 Faculty of Medicine, Sigmund Freud Private University, 1020 Vienna, Austria; 5 NUTRIM—Institute for Nutrition and Translational Research in Metabolism, 6200 Maastricht, The Netherlands; 6 Department of Internal Medicine and Pulmonology, Clinic Hietzing, Vienna Healthcare Group, 1130 Vienna, Austria; 7 Department of Respiratory and Pulmonary Diseases, Site Penzing of Clinic Ottakring, Vienna Healthcare Group, 1130 Vienna, Austria

**Keywords:** glomerular filtration rate, creatinine, body composition, metabolic syndrome, renal insufficiency, chronic

## Abstract

**Background/Objectives:** Serum creatinine (SCr)-based estimates of glomerular filtration rate (eGFR_SCr_) are influenced by muscle mass. This study investigated whether normalizing SCr to dual-energy X-ray absorptiometry (DEXA)-derived lean mass index (LMI) alters associations with cardiometabolic phenotypes. **Methods:** We analyzed 11,141 adults (mean age 46.0 ± 17.2 years) from the Austrian LEAD (Lung, hEart, sociAl, boDy) cohort. SCr was normalized to sex- and age-specific LMI reference values to calculate LMI-adjusted eGFR (eGFR_LMISCr_) within individuals. Associations with prevalent cardiometabolic conditions were compared using age- and sex-adjusted logistic regression with paired bootstrap resampling. Annualized eGFR change was compared in 7487 participants with complete follow-up measurements. **Results:** The estimates were closely correlated (R^2^ = 0.897), but LMI adjustment changed GFR-category assignment in both directions. Higher eGFR_LMISCr_ was positively associated with prevalent dyslipidemia, hypertension, pre-diabetes, diabetes, metabolic syndrome and obesity. Differences in annualized change depended on age and sex, with lower LMI-adjusted slopes in women, particularly at younger ages, and higher slopes in men. **Conclusion:** LMI adjustment influences eGFR slopes in a sex- and age-specific manner and changes both filtration function category assignment and associations with cardiometabolic phenotypes. As these associations may partly reflect the direct incorporation of body composition into the estimate, our findings will require validation against measured GFR or an independent filtration marker.

## 1. Introduction

It is now well recognized that the clinical significance of CKD extends beyond drug dosing and renal replacement therapy and that it is a potent, independent driver of cardiometabolic risk, including hypertension, dyslipidemia, and cardiovascular mortality [1,2,3,4].

The accurate assessment of glomerular filtration rate (GFR) is fundamental to the diagnosis, staging, and management of chronic kidney disease (CKD). Traditionally, estimated GFR (eGFR) derived from serum creatinine (SCr) serves as the primary tool in clinical practice due to its cost-effectiveness and accessibility. However, the reliability of SCr as a filtration marker is intrinsically limited, most notably but not exclusively by individual variations in skeletal muscle mass, which serves as the primary reservoir for creatine and its byproduct, creatinine [5,6].

Standard eGFR equations, such as the CKD-EPI (Chronic Kidney Disease Epidemiology Collaboration) 2009 and 2021 formulae, account for average differences associated with age and sex [7,8]. Despite these adjustments, substantial interindividual variation in muscle mass persists. In people with unusually high or low muscle mass, including athletes and patients with muscle wasting, creatinine-based eGFR may differ substantially from measured GFR [9,10,11]. Cystatin C provides a complementary filtration marker with different non-GFR determinants, but is often unavailable in historical cohorts. Associations between creatinine-based eGFR and cardiometabolic outcomes may therefore reflect both filtration and nonfiltration influences [12].

Several methods are available to quantify body composition, including dual-energy X-ray absorptiometry (DEXA) [13]. Lean mass index (LMI), calculated as DEXA-derived lean mass divided by height squared, provides a size-indexed measure of lean tissue [14,15]. It is a proxy for muscularity rather than a direct measurement of skeletal muscle or creatinine generation. Incorporating LMI into the interpretation of serum creatinine may alter its associations with cardiometabolic phenotypes.

Previous studies have examined the relationship between muscle mass and creatinine and the use of creatinine-related measures as markers of muscularity [9,16,17].

We hypothesized that LMI adjustment would alter the magnitude and direction of associations between eGFR and prevalent cardiometabolic conditions in a well-characterized Austrian general population cohort. We also examined agreement between estimates, GFR-category transitions, and differences in annualized eGFR change. The study was not designed to validate GFR accuracy or predict future clinical events.

## 2. Materials and Methods

### 2.1. Study Design

The study was conducted within the framework of the Austrian LEAD (Lung, hEart, sociAl, boDy) study (ClinicalTrials.gov identifier: NCT01727518), which was approved by the Ethics Committee of the City of Vienna (Ethikkommission der Stadt Wien; protocol number EK-11-117-0711). All study procedures were performed in accordance with the Declaration of Helsinki, and written informed consent was obtained from all participants and/or their legal guardians. LEAD is an ongoing longitudinal, observational, population-based cohort study [18]. The mean interval between baseline and follow-up in the present analysis was 4.25 ± 0.33 years. The final analytical cohort comprised 11,141 participants aged ≥18 years with available serum creatinine, eGFR, and DEXA-derived LMI measurements. Participants with LMI z-scores outside −5 to +5 were additionally excluded to limit the influence of extreme or implausible values.

Missing data were not imputed for the primary analyses. Participants with missing values required for a given analysis were excluded from that analysis, resulting in analysis-specific sample sizes. Whole-body DXA scans were performed using a Lunar Prodigy DEXA scanner (GE Healthcare/GE Lunar, Madison, WI, USA), with body composition analysed using enCORE software version 18. Quality control and calibration were performed according to the manufacturer’s recommendations. A phantom scan was performed before the first measurement of each day. Participant positioning was standardized and verified by trained study personnel before measurement, and scan quality was subsequently reviewed. Serum creatinine was measured using an enzymatic assay on a Siemens Dimension VISTA 1500 (Siemens Healthineers, Munich, Germany) system in an ISO-certified laboratory. Calibration and quality control were performed according to the manufacturer’s recommendations. DXA and blood sampling were always performed on the same day (blood sampling first) [18]. Serum creatinine concentrations are expressed in mg/dL for calculation of eGFR with the CKD-EPI 2009 equation [7].

For the longitudinal analyses, participants were required to have serum creatinine and LMI measurements at both visits. Of the 11,141 participants in the cross-sectional analytical cohort, 7487 (67.20%) had participated in the follow-up measurements required for the longitudinal analysis; 3654 (32.80%) were lost to follow-up and did not contribute to that analysis. The longitudinal results therefore describe the subset with complete paired measurements.

### 2.2. Measurements

Lean-mass-adjusted creatinine was derived to account for interindividual variation in lean tissue. Each participant was assigned a sex- and age-specific reference LMI at the 50th percentile using previously established LEAD-derived body-composition reference values for each visit [19]. The median was selected to represent typical LMI for age and sex while limiting the influence of extreme observations. Individual serum creatinine concentrations were normalized to the corresponding reference LMI using the following formula:

$$\mathrm{LMISCr}=\mathrm{Serum}\frac{\mathrm{Creatinine}\times \mathrm{LMI}\;\mathrm{of}\;50\mathrm{th}\;\mathrm{percentile}\;\mathrm{of}\;\mathrm{age}\wedge \mathrm{sex}-\mathrm{specific}\;\mathrm{LMI}\;\mathrm{percentile}}{\mathrm{LMI}\;\mathrm{of}\;\mathrm{individual}\;\mathrm{participant}}\mathrm{LMISCr}$$

Thus, no adjustment was applied when an individual’s LMI equalled the age- and sex-specific median. Creatinine was proportionally reduced for LMI above the reference and increased for LMI below it. This transformation assumes that, at a given GFR and with other determinants held constant, creatinine generation scales proportionally with LMI [9,16]. The transformed concentration is therefore a model-based normalization.

eGFR was calculated using both raw and LMI-adjusted serum creatinine with the CKD-EPI 2009 equation [7]. For categorical analyses, eGFR values were classified according to Kidney Disease: Improving Global Outcomes (KDIGO) GFR categories, with a collapsed five-category classification, combining the two lowest eGFR strata due to small sample sizes. Complete eGFR strata are included in descriptive statistics and reclassification analysis. For all analyses, a five-category grouping was defined as: G1 (≥90 mL/min/1.73 m^2^), G2 (60–<90 mL/min/1.73 m^2^), G3a (45–<60 mL/min/1.73 m^2^), G3b (30–<45 mL/min/1.73 m^2^), and G4/5 (<30 mL/min/1.73 m^2^) [5]. For regression modelling, eGFR categories were encoded with ≥90 mL/min/1.73 m^2^ as the reference group.

Height and weight were measured using a calibrated stadiometer and precision scale and used to calculate body mass index (BMI, kg/m^2^). Waist circumference was also measured and used to calculate waist-to-height ratio. Fat mass index (FMI) was obtained from DEXA scans. Age, smoking status, and cumulative smoking exposure were collected using standardized, interviewer-administered questionnaires.

Metabolic syndrome was defined according to International Diabetes Federation (IDF) criteria, based on central obesity, triglyceride levels, HDL cholesterol, blood pressure and fasting glucose [20,21]. Dyslipidemia was defined as at least one of the following: LDL-C ≥ 100 mg/dL, triglycerides ≥ 150 mg/dL, total cholesterol ≥ 200 mg/dL, sex-specific low HDL-C or statin use. Obesity was defined based on the Lancet Diabetes and Endocrinology Commission (Obesity_LDEC_) or WHO (Obesity_WHO_; BMI ≥ 30 kg/m^2^) definitions: participants were classified as having Obesity_LDEC_ if they had BMI ≥ 40 kg/m^2^; BMI ≥ 30 kg/m^2^ in combination with either a waist-to-height ratio ≥ 0.50 or sex-specific waist circumference thresholds (≥102 cm in men or ≥88 cm in women); or BMI < 30 kg/m^2^ in combination with both elevated waist-height ratio and sex-specific waist circumference thresholds [22,23]. Pre-diabetes and diabetes were classified using fasting plasma glucose and HbA1c thresholds [24], and hypertension was defined as systolic blood pressure ≥ 140 mmHg, diastolic blood pressure ≥ 90 mmHg, or current use of anti-hypertensive medication. Cardiovascular disease was defined as self-reported physician-diagnosed myocardial infarction, coronary artery disease or heart failure.

### 2.3. Statistical Analysis

All statistical analyses were performed in R (version 4.3.0; R Foundation for Statistical Computing, Vienna, Austria), with statistical significance defined as *p* < 0.05. Data are presented as mean ± standard deviation (SD) or *n* (%). Group comparisons across GFR categories used one-way ANOVA or t-tests for continuous variables and χ^2^ tests for categorical variables. Age and sex associations with continuous eGFR were assessed using linear regression, whereas associations with GFR-category membership were assessed using logistic regression; both included an age-by-sex interaction. Regression *p*-values were obtained using Wald tests. GFR-category transitions were summarized as counts and visualized in an alluvial plot, without assigning correctness to either estimate.

For regression analyses, raw and LMI-adjusted eGFR were standardized separately to a mean of 0 and standard deviation of 1; thus, odds ratios represent the change in odds associated with a one-standard-deviation higher eGFR. Logistic regression models were adjusted for age and sex a priori. These covariates were selected as common determinants of serum creatinine, LMI and eGFR and were included to provide a common minimal adjustment set when comparing the two eGFR definitions. Measures of body size or adiposity were not included in the primary models because they are closely related to LMI and, for some outcomes, form part of the outcome definition itself.

To directly compare associations obtained using raw and LMI-adjusted eGFR, the regression coefficients for the two eGFR variables were compared on the log-odds scale. For each outcome, the difference was defined as Δlog(OR) = log(OR_LMI-adjusted) − log(OR_raw), with the corresponding ratio of odds ratios calculated as exp[Δlog(OR)] = OR_LMI-adjusted/OR_raw. A ratio of 1 indicates equal estimated ORs; values > 1 or <1 indicate numerically higher or lower ORs, respectively, for LMI-adjusted eGFR. Whether an association is stronger, attenuated or reversed must be assessed from the individual ORs relative to the null value of 1, rather than from the ratio alone.

Differences between associations were evaluated using a nonparametric participant-level bootstrap with 1000 resamples. For each bootstrap sample, the two logistic regression models were refitted using the same resampled participants and the difference between the corresponding eGFR coefficients was calculated. Bootstrap confidence intervals for the ratio of odds ratios were obtained by exponentiating the corresponding percentile confidence limits for Δlog(OR). *p* values were calculated using empirical tail-counting. Inferences about differences between associations were based on these paired bootstrap comparisons, rather than on overlap between confidence intervals from the separate models.

Agreement between conventional creatinine-based eGFR and LMI-adjusted eGFR was assessed to quantify the magnitude and variability of the change introduced by LMI adjustment. Because both estimates were derived from serum creatinine using the same CKD-EPI equation, neither was considered a reference standard for measured renal function. Agreement was therefore interpreted as similarity between the two estimation approaches rather than validation or diagnostic accuracy. Pearson correlation and Bland–Altman analysis were used to assess the relationship and distribution of individual differences between estimates. Mean difference and 95% limits of agreement were calculated. The proportions of LMI-adjusted estimates falling within 10% (P10) and 30% (P30) of the conventional eGFR estimate were additionally reported as relative-difference measures, indicating the extent to which LMI adjustment altered the conventional estimate. Proportional bias was assessed by regressing the difference between estimates on their mean, and associations between the difference and LMI were examined using linear regression, including an LMI-by-sex interaction term.

Individual eGFR slopes were calculated for participants with complete and valid serum creatinine measurements at both study visits (*n* = 7487). Annualized eGFR change was derived as the difference in eGFR between visits divided by the time interval and was computed separately for eGFR based on raw and LMI-adjusted creatinine. For the purpose of the longitudinal analysis, both eGFR_SCr_ and eGFR_LMISCr_ were calculated for each individual timepoint, i.e., from repeat serum creatinine and DEXA measurements. To compare longitudinal decline between raw and LMI-adjusted eGFR, slopes were assessed in a mixed-effects linear model including eGFR definitions, age, and sex, with a random intercept for participant to account for within-person pairing.

### 2.4. Data Availability Statement

Due to institutional agreements and ongoing data collection activity, unrestricted access cannot be granted to our primary data. However, data specifically pertaining to this manuscript may be made available upon reasonable request made to the corresponding author.

### 2.5. Use of Generative AI Statement

During the preparation of this work the authors used Google Gemini 3 to improve sentence structure in some instances. After using this tool, the authors reviewed and edited the content as needed and take full responsibility for the content of the published article.

## 3. Results

The study cohort comprised 11,141 adults with a mean age of 46.0 ± 17.2 years, of whom 47.3% were male. A high percentage of included subjects exhibited cardiometabolic risk factors, including dyslipidemia (84.2%), metabolic syndrome (26.5%), pre-diabetes or diabetes (26.1%), and hypertension (17.0%). A summary of baseline characteristics of the total cohort and by GFR category is provided in Table 1. The full side-by-side comparison of group characteristics between eGFR_SCr_ and eGFR_LMISCr_ is provided in Table S1. A flow chart containing information on study participants included in the cross-sectional and longitudinal analyses is provided in Figure S1. Histograms of the eGFR distribution stratified by estimation method are provided in Figure S2.

Paired bootstrap comparisons of standardized, age- and sex-adjusted logistic regression coefficients showed differences between eGFR_LMISCr_ and eGFR_SCr_ for all outcomes (all *p* < 0.001; all 95% confidence intervals for the ratios of ORs excluded 1; Table 2 and Figure S3). LMI-adjusted eGFR showed positive associations with dyslipidemia, pre-diabetes, diabetes and metabolic syndrome. For hypertension, the association changed from inverse to positive (OR 0.82 versus 1.27). For cardiovascular disease, the inverse association with eGFR_SCr_ (OR 0.84) was attenuated to a near-null association with eGFR_LMISCr_ (OR 1.04, 95% CI 0.93–1.17; ratio of ORs 1.23, bootstrap 95% CI 1.16–1.31). Additionally, eGFR_LMISCr_ showed stronger positive associations with obesity according to both WHO and LDEC Commission criteria [22,23] (Obesity_WHO_ and Obesity_LDEC_, respectively).

**Figure 1:**
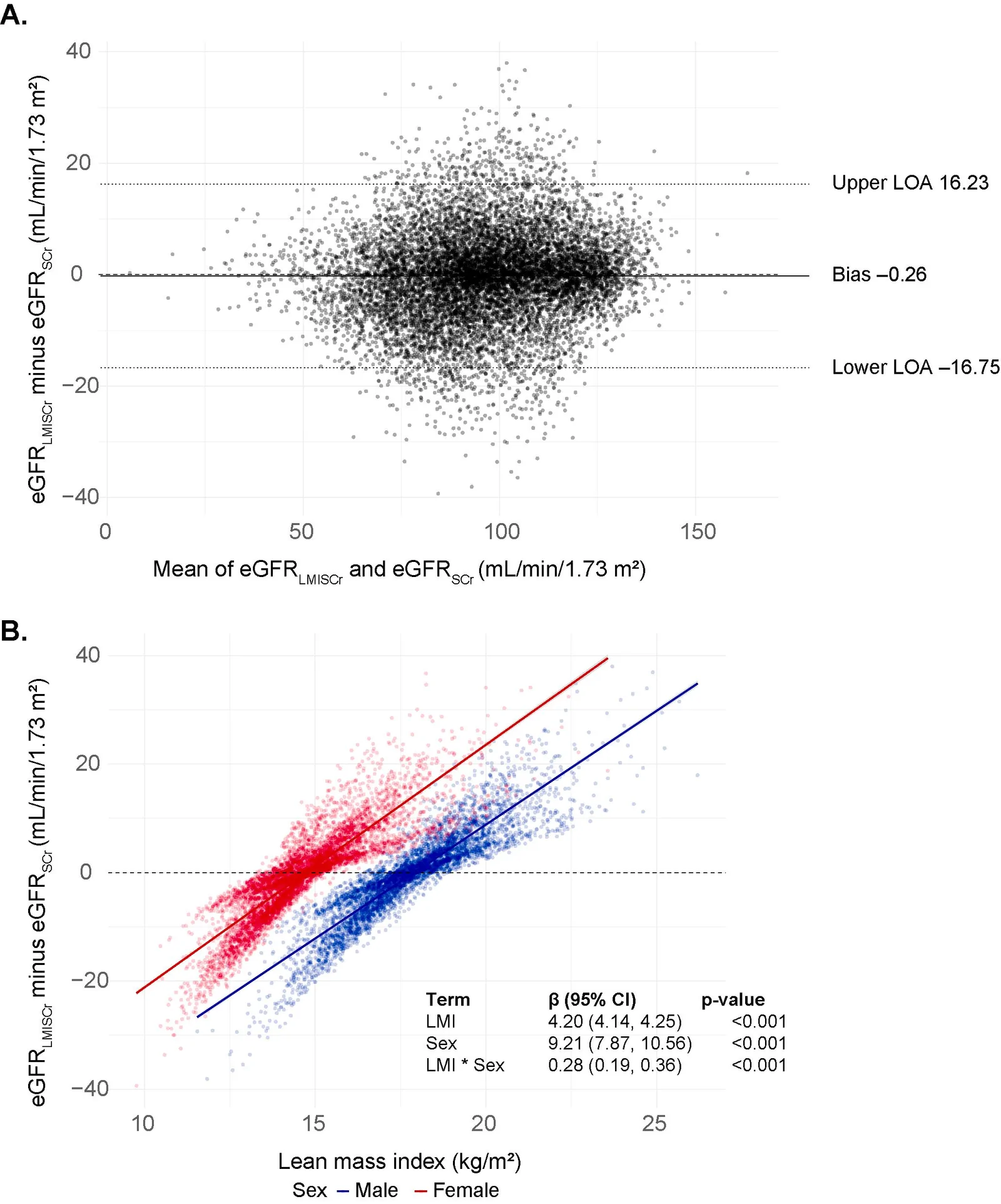
Agreement between eGFR_SCr_ and eGFR_LMISCr_ and differences according to lean mass index (LMI) and sex. Figure legend: (**A**) Bland–Altman plot showing the difference between LMI-adjusted and conventional eGFR against their mean. The solid horizontal line represents the mean bias, and the dashed horizontal lines represent the 95% limits of agreement (LOA). (**B**) Difference between LMI-adjusted and conventional eGFR according to LMI, stratified by sex. Lines represent fitted linear regression models with 95% confidence intervals. Sex was coded with males as the reference category. The regression model included LMI, sex, and the LMI × sex interaction.

Comparison between standard eGFR_SCr_ and eGFR_LMISCr_ showed a high correlation (R^2^ = 0.897). The mean difference revealed a negligible fixed bias of −0.26 mL/min/1.73 m^2^ with an SD of 8.41 and 95% limits of agreement (LoA) from −16.75 to 16.23 mL/min/1.73 m^2^ (Figure 1A). Relative-difference analyses showed that 75.2% of estimates were within 10% (P10) and 99.2% were within 30% (P30) of the reference raw eGFR. A small proportional difference was observed (slope: −0.057, *p* < 0.001), indicating that individual differences were more pronounced at certain levels of kidney function. The difference between eGFR_SCr_ and eGFR_LMISCr_ additionally showed an association between LMI and eGFR difference (β = 4.20) with evidence of interaction between LMI and sex (β = 0.28; Figure 1B). Overall, the equations showed minimal systematic difference but relevant individual variability.

The distribution of eGFR in the total population remained approximately normal under both definitions (Figure S2). Cross-sectional eGFR decreased with increasing age under both definitions (Figure S4). Age and sex associations with continuous eGFR and GFR-category membership are summarized descriptively in Table 3 and Table 4, respectively.

The application of lean mass adjustment resulted in shifts in the classification of chronic kidney disease (CKD) stages, with the highest relative number of reclassifications occurring between stages G1 and G2 as well as G3a and G2 (Table 5 and Figure S5). Overall, reclassifications were more likely in individuals at the extremes of LMI Z-score (Tables S2 and S3).

**Figure 2:**
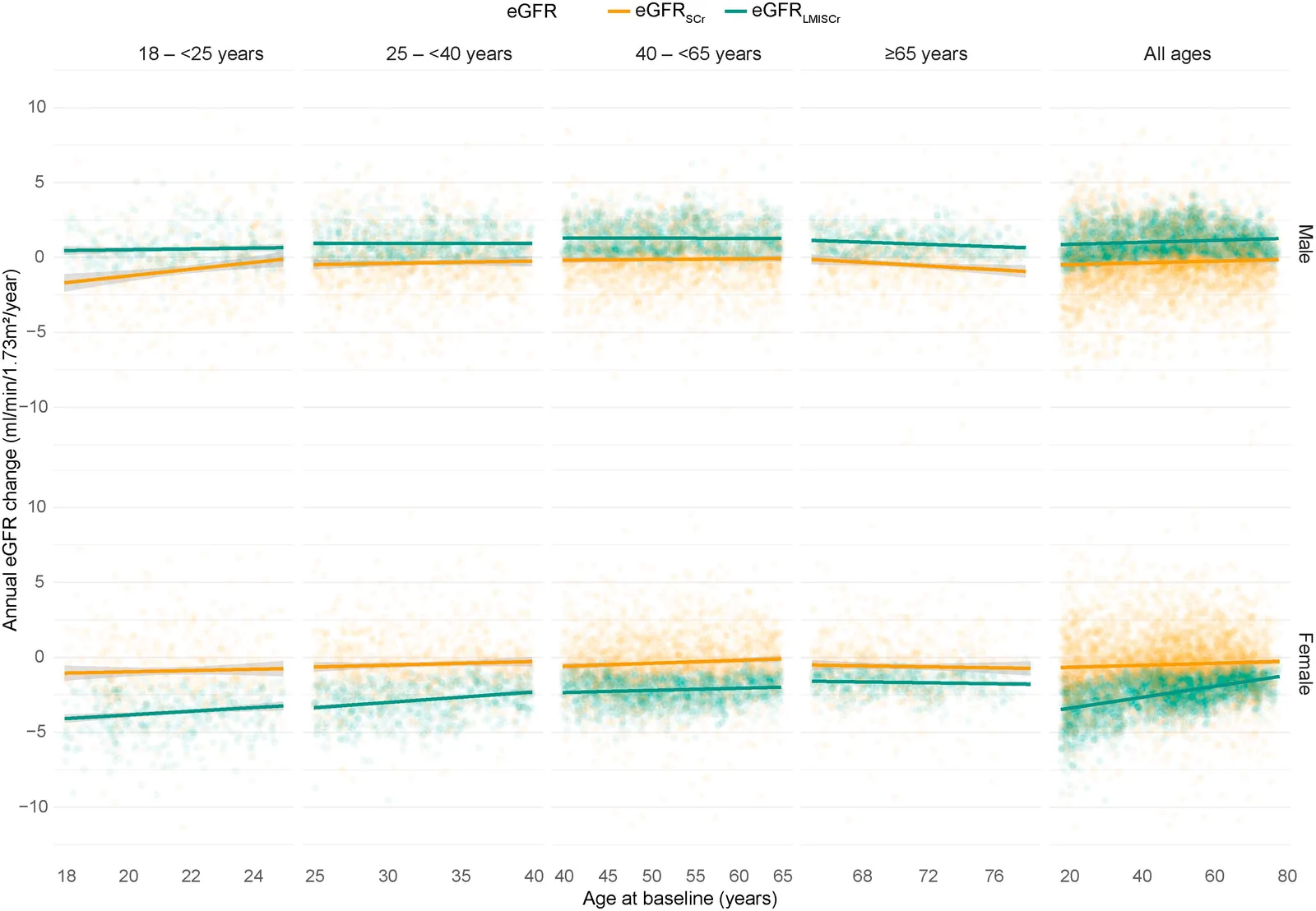
Annualized change in eGFR stratified by age bins and sex. Figure legend: Regression models are shown as estimates and 95% confidence intervals (CIs) obtained from linear mixed-effects regression models assessing annualized change in eGFR. Individual participants were included as random intercepts to account for repeated measurements, with individual data points presented as jitter plots.

Longitudinally, eGFR_LMISCr_ showed significantly greater yearly decline than eGFR_SCr_ (Table S4 and Figure S6). This was largely driven by a faster decline of eGFR_LMISCr,_ particularly in young women, with a significant effect of sex and age (Table 6 and Figure 2). In sex-stratified analyses, eGFR_LMISCr_ was positively associated with metabolic syndrome, pre-diabetes, diabetes and both obesity definitions in men and women. Associations of eGFR_LMISCr_ with cardiovascular disease, hypertension and dyslipidemia were statistically significant in women but not in men (Table S5).

## 4. Discussion

In this large population-based cohort, adjustment of serum creatinine for DEXA-derived LMI strengthened associations with several cardiometabolic phenotypes and altered annualized eGFR change in an age- and sex-dependent manner.

The biological rationale for our approach is supported by the relationship between lean mass and creatinine generation; however, reports on previous attempts to normalize measured creatinine to other determinants of muscle mass are sparse. Studies in healthy adults have linked lean mass to serum and urinary creatinine [16], and Nankivell et al. demonstrated muscle-associated eGFR error against measured GFR in 137 kidney transplant recipients [9]. Kim et al. used creatinine and cystatin C to estimate muscle mass, illustrating the information about muscularity contained in creatinine when filtration is considered [25]. Our study examines the converse question: whether incorporating measured lean mass changes the epidemiological information obtained from creatinine-based eGFR. The age- and sex-specific median provides an interpretable reference for this exploration, while proportional scaling remains a modelling assumption. In CRIC, adding bioimpedance phase angle and other variables did not materially improve GFR prediction beyond a model already containing creatinine and cystatin C [26]. That finding underscores that the potential benefit of adjustments for lean mass depends on the available markers and study population. Our study adds novelty by reporting on associations of DEXA-measured lean mass in a predominantly preserved-eGFR general population cohort.

Several limitations define the interpretation of our data. Importantly, we solely report associations which demonstrate that LMI-adjustment of serum creatinine may be of scientific interest pending confirmatory validation trials, particularly in population research. The absence of measured GFR and cystatin C prevents assessment of whether LMI adjustment improves true filtration accuracy and validation against measured GFR, or at minimum an independent filtration marker, is required before application [5,27]. DEXA-derived LMI is a proxy for creatinine-producing tissue, and neither the proportional transformation nor the cohort-derived reference values have been independently validated for this purpose. Residual confounding by adiposity and other metabolic determinants remains possible in the age- and sex-adjusted models, and associations with prevalent conditions do not establish causality or prospective prediction. Albuminuria was unavailable, limiting renal phenotyping and CKD classification; while a recent report from a neighboring European country suggests a prevalence of albuminuria of roughly 10% in the general population, the true prevalence in our cohort cannot be inferred from the general population setting [28]. Estimates in advanced GFR categories are particularly uncertain because G4/5 included only 10 and seven participants under the respective definitions. Finally, incomplete paired follow-up in 32.8% of the analytical cohort may introduce selection bias. Nevertheless, our findings support the scientific value of incorporating measured body composition when investigating creatinine-based eGFR in principle, particularly in cohorts where DEXA data are available but alternative filtration markers are not.

The predominance of participants with eGFR ≥ 60 mL/min/1.73 m^2^ places our findings primarily in the context of population epidemiology. Differences within this range remain relevant when studying continuous associations with metabolic health, even when they do not necessarily imply a clinical diagnosis. The small mean difference between estimates (−0.26 mL/min/1.73 m^2^) coexisted with limits of agreement from −16.75 to 16.23 mL/min/1.73 m^2^, demonstrating that population averages can obscure appreciable and potentially clinically relevant individual differences. Reclassification was bidirectional: 772 participants moved from G1 to G2 and 690 from G2 to G1, while 156 participants originally in G2 (4.09%) moved to G3a. The total number in G2 was essentially unchanged. These findings identify where body-composition adjustment affects classification without establishing which estimate is correct [5].

Longitudinal analysis adds a further dimension to the study. Over approximately 4.25 years, LMI-adjusted slopes were higher in men and lower in women than conventional slopes, with the greatest divergence in younger women. These differences show that incorporating repeated body-composition measurements can change the apparent pattern of eGFR change, even when cross-sectional age associations remain broadly similar. This is relevant to studies of ageing or metabolic change, in which changes in lean mass may influence creatinine independently of filtration [9,16]. The observed sex- and age-dependent pattern identifies younger women and participants with changing body composition as priorities for further validation. It does not yet identify a subgroup in which LMI-adjusted eGFR is a more reliable predictor. Each slope was derived from two visits and incorporates changes in creatinine, LMI and age-dependent terms; their separate contributions were not quantified. We therefore interpret the findings as differences in estimated longitudinal change, without inferring greater precision of estimation.

The particularly pronounced association with WHO-defined obesity illustrates both the sensitivity of the approach to body composition and the need for careful interpretation. The OR increased from 0.97 with conventional eGFR to 4.25 with eGFR_LMISCr_. At fixed creatinine, age and sex, a higher individual LMI lowers the normalised creatinine concentration and raises adjusted eGFR. Because LMI is related to BMI and adiposity [19,29], this transformation can introduce mathematical and biological coupling with obesity. Related pathways may also contribute to the associations with metabolic syndrome, glucose and lipid abnormalities. Age and sex adjustment does not fully separate these effects. Accordingly, the obesity result is informative about the consequences of incorporating lean mass but cannot by itself distinguish better representation of filtration from the addition of body-composition information.

These findings have a practical application in epidemiological research. Although changing the creatinine input necessarily changes eGFR, the magnitude of this change across participants and its consequences for observed metabolic associations and longitudinal comparisons are empirical questions. Our study quantifies these consequences for prevalent cardiometabolic risk factors. Population studies showing that creatinine and cystatin C have different relationships with muscle mass and mortality reinforce the potential importance for creatinine-based associations [10,12]. In historical cohorts with contemporaneous creatinine and DEXA measurements, LMI adjustment could provide an exploratory sensitivity analysis alongside conventional eGFR when alternative filtration markers cannot be obtained retrospectively. Such comparisons could help identify conclusions that depend on the representation of lean mass, particularly in participants with LMI far from age- and sex-specific reference values or with changing body composition. This use would add an explicit assessment of body-composition dependence to existing cohort analyses, while subsequent validation could determine whether it also improves GFR estimation or prospective risk prediction.

A major strength of this study is the LEAD cohort, which combines a large population-based sample with DEXA-derived body-composition measurements and detailed cardiometabolic phenotyping [18,19]. Comparing both eGFR definitions within the same participants reduces the influence of differences in sample composition, and repeated measurements in 7487 participants permit examination of longitudinal as well as cross-sectional patterns. The adjustment is based on an explicit physiological assumption and age- and sex-specific reference values, without fitting its coefficients to the cardiometabolic outcomes under investigation.

In conclusion, incorporating DEXA-derived LMI into creatinine-based eGFR reveals stronger associations with selected cardiometabolic phenotypes and different longitudinal patterns across age and sex. The study provides evidence that body composition can materially influence the epidemiological interpretation of creatinine-based estimates even when average eGFR changes little. Pending validation against measured GFR, our method could potentially offer a practical approach for further research using existing creatinine and DEXA data in historic cohorts where independent filtration markers are unavailable. Establishing its incremental value for GFR accuracy and clinical risk prediction is the next step towards translating these findings into potential scientific and clinical use.

## Figures and Tables

**Table 1:** Baseline characteristics of included participants stratified by eGFR categories.

	Total	eGFR_SCr_						eGFR_LMISCr_					
		G1	G2	G3a	G3b	G4/5	*p*	G1	G2	G3a	G3b	G4/5	*p*
**Total N (%)**	11,141	6993 (62.8)	3812 (34.2)	264 (2.4)	62 (0.6)	10 (0.1)		6910 (62.0)	3810 (34.2)	353 (3.2)	61 (0.5)	7 (0.1)	
**Males**	5265 (47.3)	3338 (47.7)	1764 (46.3)	122 (46.2)	35 (56.5)	6 (60.0)	0.289	3261 (47.2)	1806 (47.4)	157 (44.5)	37 (60.7)	4 (57.1)	0.214
**Creatinine, mg/dL**	0.84 ± 0.19	0.78 ± 0.13	0.93 ± 0.14	1.17 ± 0.16	1.56 ± 0.27	2.92 ± 1.97	<0.001	0.78 ± 0.14	0.91 ± 0.14	1.11 ± 0.18	1.59 ± 0.33	3.24 ± 2.31	<0.001
**Creatinine_LMI_, mg/dL**	0.84 ± 0.20	0.78 ± 0.15	0.92 ± 0.16	1.15 ± 0.19	1.52 ± 0.31	2.69 ± 1.94	<0.001	0.76 ± 0.14	0.94 ± 0.15	1.18 ± 0.17	1.58 ± 0.24	3.23 ± 2.14	<0.001
**eGFR_SCr_ (mL/min/1.73 m^2^)**	96.15 ± 19.11	107.83 ± 11.99	78.71 ± 7.89	54.59 ± 3.98	39.71 ± 3.91	22.82 ± 7.85	<0.001	106.86 ± 13.44	81.19 ± 10.63	59.02 ± 8.30	40.09 ± 6.69	21.71 ± 9.93	<0.001
**eGFR_LMISCr_ (mL/min/1.73 m^2^)**	95.89 ± 19.96	106.90 ± 14.12	79.51 ± 11.57	56.23 ± 7.38	41.33 ± 6.49	26.71 ± 11.01	<0.001	108.43 ± 12.35	78.02 ± 7.97	54.58 ± 3.87	39.50 ± 3.94	20.76 ± 8.49	<0.001
**LMI, kg/m^2^**	16.2 ± 2.3	16.0 ± 2.3	16.5 ± 2.3	16.7 ± 2.0	17.2 ± 2.2	18.8 ± 2.8	<0.001	16.4 ± 2.4	15.8 ± 2.1	15.4 ± 2.0	16.8 ± 2.7	16.6 ± 3.1	<0.001
**BMI, kg/m^2^**	25.7 ± 4.8	25.0 ± 4.7	26.8 ± 4.6	28.8 ± 4.7	29.4 ± 4.8	31.9 ± 4.5	<0.001	25.8 ± 5.1	25.4 ± 4.2	25.9 ± 4.4	28.2 ± 4.7	27.9 ± 5.8	<0.001
**Metabolic syndrome**	2947 (26.5)	1215 (17.4)	1506 (39.5)	172 (65.2)	45 (72.6)	9 (90.0)	<0.001	1420 (20.5)	1296 (34.0)	183 (51.8)	42 (68.9)	6 (85.7)	<0.001
**Obesity_WHO_**	1888 (16.9)	955 (13.7)	802 (21.0)	97 (36.7)	26 (41.9)	8 (80.0)	<0.001	1303 (18.9)	500 (13.1)	59 (16.7)	22 (36.1)	4 (57.1)	<0.001
**Obesity_LDEC_**	4823 (43.3)	2367 (33.8)	2202 (57.8)	195 (73.9)	50 (80.6)	9 (90.0)	<0.001	2731 (39.5)	1846 (48.5)	196 (55.5)	46 (75.4)	4 (57.1)	<0.001
**Dyslipidemia**	8168 (84.2)	4494 (79.0)	3369 (91.7)	237 (90.8)	58 (93.5)	10 (100.0)	<0.001	4491 (79.8)	3291 (90.1)	322 (92.5)	57 (93.4)	7 (100.0)	<0.001
**Pre-diabetes/diabetes**	2900 (26.1)	1303 (18.7)	1392 (36.7)	159 (60.7)	38 (62.3)	8 (80.0)	<0.001	1394 (20.3)	1294 (34.2)	173 (49.1)	35 (58.3)	4 (57.1)	<0.001
**Pre-diabetes**	2304 (20.8)	1056 (15.2)	1111 (29.3)	110 (42.0)	20 (32.8)	7 (70.0)	<0.001	1103 (16.0)	1046 (27.6)	131 (37.2)	20 (33.3)	4 (57.1)	<0.001
**Diabetes**	596 (5.3)	247 (3.5)	281 (7.4)	49 (18.6)	18 (29.0)	1 (10.0)	<0.001	291 (4.2)	248 (6.5)	42 (11.9)	15 (24.6)	0 (0.0)	<0.001
**Cardiovascular disease**	804 (7.2)	197 (2.8)	494 (13.0)	76 (28.8)	31 (50.0)	6 (60.0)	<0.001	229 (3.3)	458 (12.0)	78 (22.1)	35 (57.4)	4 (57.1)	<0.001
**Hypertension**	1890 (17.0)	553 (7.9)	1128 (29.6)	152 (57.6)	48 (77.4)	9 (90.0)	<0.001	675 (9.8)	1010 (26.5)	154 (43.6)	46 (75.4)	5 (71.4)	<0.001

Data are presented as n (%) or mean (standard deviation). eGFR categories were defined according to the serum creatinine-based eGFR (eGFR_SCr_) and the lean-mass-index-adjusted serum creatinine-based eGFR (eGFR_LMISCr_). *p*-values indicate differences across eGFR categories. Significance was considered at *p* < 0.05. Abbreviations: LMI, lean mass index; BMI, body mass index; eGFR, estimated glomerular filtration rate; eGFR_LMISCr_, lean-mass-index-adjusted serum creatinine-based estimated glomerular filtration rate; eGFR_SCr_, serum creatinine-based estimated glomerular filtration rate.

**Table 2:** Associations of eGFR with comorbidities.

Outcomes	eGFR_SCr_		eGFR_LMISCr_			
	OR (95% CI)	*p*-Value	OR (95% CI)	*p*-Value	∆ OR (95% CI)	*p*-Value
Cardiovascular disease	0.84 (0.75, 0.95)	0.004	1.04 (0.93, 1.17)	0.499	1.23 (1.16, 1.31)	<0.001
Hypertension	0.82 (0.75, 0.89)	<0.001	1.27 (1.16, 1.39)	<0.001	1.55 (1.48, 1.63)	<0.001
Metabolic syndrome	0.95 (0.88, 1.02)	0.157	1.69 (1.57, 1.82)	<0.001	1.78 (1.70, 1.87)	<0.001
Dyslipidemia	1 (0.92, 1.09)	0.96	1.19 (1.1, 1.29)	<0.001	1.19 (1.12, 1.25)	<0.001
Prediabetes	1.05 (0.97, 1.14)	0.203	1.31 (1.21, 1.41)	<0.001	1.24 (1.18, 1.31)	<0.001
Diabetes	1.21 (1.05, 1.39)	0.007	2.22 (1.91, 2.58)	<0.001	1.83 (1.68, 2.01)	<0.001
Obesity_LDEC_	1.04 (0.97, 1.1)	0.278	2.56 (2.39, 2.75)	<0.001	2.48 (2.37, 2.59)	<0.001
Obesity_WHO_	0.97 (0.9, 1.05)	0.402	4.25 (3.84, 4.71)	<0.001	4.39 (4.07, 4.78)	<0.001

Estimates are odds ratios (OR) and 95% confidence intervals (CI) obtained from logistic regression models adjusted for age and sex. ΔOR represents the bootstrap-derived ratio comparing the OR obtained using eGFR_LMISCr_ with that obtained using eGFR_SCr_; a ratio of 1 indicates equal estimated ORs, and values > 1 or <1 indicate numerically higher or lower ORs, respectively, for eGFR_LMISCr_. Association strength and direction must be interpreted from the individual ORs relative to 1. ORs represent the change in odds per 1-SD higher eGFR. Conventional and LMI-adjusted eGFR were standardised separately. Confidence intervals and *p*-values for ΔOR were obtained from 1000 paired participant-level bootstrap resamples. For this paired comparison, the null ratio is 1. Statistical significance was defined as *p* < 0.05.

**Table 3:** Age and sex effects on eGFR.

	eGFR_SCr_			eGFR_LMISCr_		
	Unstandardised β (95% CI)	Standardised β (95% CI)	*p*-Value	Unstandardised β (95% CI)	Standardised β (95% CI)	*p*-Value
Female	3.13 (1.79, 4.47)	0.01 (0, 0.02)	<0.001	2.97 (1.5, 4.43)	0.01 (0, 0.03)	<0.001
Age	−0.80 (−0.82, −0.78)	−0.75 (−0.76, −0.74)	<0.001	−0.81 (−0.83, −0.79)	−0.72 (−0.73, −0.71)	<0.001
Female × Age	−0.06 (−0.09, −0.03)	−0.03 (−0.04, −0.01)	<0.001	−0.05 (−0.08, −0.02)	−0.02 (−0.04, −0.01)	<0.001

Estimates (β) and 95% confidence intervals (CIs) obtained from linear regression models assessing the associations of age, sex, and the age-by-sex interaction. Female sex was compared with male sex as the reference category. Models were fitted separately for creatinine-based eGFR (eGFR_SCr_) and lean-mass-index-adjusted creatinine-based eGFR (eGFR_LMISCr_).

**Table 4:** Age and sex effects on eGFR, stratified by eGFR-strata.

eGFR Strata	eGFR_SCr_						eGFR_LMISCr_					
	Age	*p*-Value	Female	*p*-Value	Age × Female	*p*-Value	Age	*p*-Value	Female	*p*-Value	Age × Sex	*p*-Value
G2	1.09 (1.09, 1.1)	<0.001	0.82 (0.56, 1.2)	0.308	1 (1, 1.01)	0.182	1.09 (1.08, 1.09)	<0.001	0.96 (0.68, 1.37)	0.805	1 (0.99, 1.01)	0.985
G3a	1.21 (1.18, 1.24)	<0.001	0.31 (0.03, 3.08)	0.316	1.02 (0.99, 1.05)	0.242	1.18 (1.15, 1.2)	<0.001	0.29 (0.05, 1.67)	0.165	1.02 (0.99, 1.05)	0.129
G3b	1.23 (1.19, 1.27)	<0.001	0 (0, 0)	<0.001	1.09 (1.08, 1.1)	<0.001	1.21 (1.17, 1.25)	<0.001	0.01 (0, 0.01)	<0.001	1.07 (1.06, 1.08)	<0.001
G4/5	1.29 (1.27, 1.3)	<0.001	0 (0, 0)	<0.001	1.16 (1.14, 1.18)	<0.001	1.36 (1.36, 1.40)	<0.001	†	<0.001	0.86 (0.85, 0.88)	<0.001

Estimates are odds ratios (ORs) and 95% confidence intervals (CIs) obtained from logistic regression models assessing the associations of age, sex, and the age-by-sex interaction with categorical estimated filtration function. Categories were defined as G1 (≥90 mL/min/1.73 m^2^), G2 (60–<90 mL/min/1.73 m^2^), G3a (45–<60 mL/min/1.73 m^2^), G3b (30–<45 mL/min/1.73 m^2^), and G4/5 (<30 mL/min/1.73 m^2^). Female sex was compared with male sex as the reference category. Models were fitted separately for creatinine-based eGFR (eGFR_SCr_) and lean-mass-index-adjusted creatinine-based eGFR (eGFR_LMISCr_). † Estimate could not be reasonably estimated because of numerical instability/separation, with the estimated OR exceeding 10^2^. Significance was considered at *p* < 0.05.

**Table 5:** Reclassification of eGFR-strata according to creatinine-based and lean-mass-index-adjusted creatinine-based eGFR.

eGFR_SCr_	eGFR_LMISCr_					Total n in eGFR_SCr_
	G1	G2	G3a	G3b	G4/5	
**G1**	6220	772	1	0	0	6993
**G2**	690	2966	156	0	0	3812
**G3a**	0	71	183	10	0	264
**G3b**	0	1	13	46	2	62
**G4/5**	0	0	0	5	5	10
**Total n in eGFRLMISCr**	6910	3810	353	61	7	11,141

eGFR_SCr_ denotes creatinine-based estimated glomerular filtration rate, and eGFR_LMISCr_ denotes estimated glomerular filtration rate calculated using lean-mass-index-adjusted serum creatinine. eGFR strata were defined as G1 (≥90 mL/min/1.73 m^2^), G2 (60–<90 mL/min/1.73 m^2^), G3a (45–<60 mL/min/1.73 m^2^), G3b (30–<45 mL/min/1.73 m^2^), and G4/5 (<30 mL/min/1.73 m^2^).

**Table 6:** Associations of age, sex and eGFR definitions with annualized eGFR slopes.

	β (95% CI)	*p*-Value
eGFR_SCr_ (ref. eGFR_LMISCr_)	−1.38 (−1.46, −1.3)	<0.001
Age	0.01 (0, 0.01)	<0.001
Female (ref. Male)	−3.47 (−3.55, −3.39)	<0.001
eGFR_SCr_ × Age	0 (−0.01, 0)	0.721
eGFR_SCr_ × Female	3.31 (3.2, 3.43)	<0.001
Age × Female	0.03 (0.02, 0.03)	<0.001
eGFR_SCr_ × Age × Female	−0.03 (−0.04, −0.02)	<0.001

Estimates are regression coefficients (β) and 95% confidence intervals (CIs) obtained from linear mixed-effects regression models assessing annualized change in eGFR. Individual participants were included as random intercepts to account for repeated measurements. Coefficients represent the difference in eGFR change per year (mL/min/1.73 m^2^/year), unless otherwise specified. The analysis included 7487 participants. eGFR_LMISCr_ was used as the reference eGFR definition and male sex as the reference category. Statistical significance was considered at *p* < 0.05.

## Data Availability

Due to institutional agreements and ongoing data collection activity, unrestricted access cannot be granted to our primary data. However, data specifically pertaining to this manuscript may be made available upon reasonable request made to the corresponding author.

## Supplementary Materials

The following supporting information can be downloaded at: https://www.mdpi.com/article/10.3390/diagnostics16213540/s1. Figure S1: Study population flow chart; Figure S2: Distribution of eGFR in the total population; Figure S3: Comparisons of outcome associations between eGFR definitions; Figure S4: Distribution of eGFR by age and sex; Figure S5: Transitions between GFR categories by eGFR definition; Figure S6: Annualized change in eGFR; Table S1: Baseline characteristics of included participants stratified by eGFR categories; Table S2: Reclassification between raw and LMI-adjusted CKD-EPI 2009 eGFR categories according to LMI percentile range; Table S3: Overall reclassification between raw and LMI-adjusted CKD-EPI 2009 eGFR categories across LMI percentile ranges; Table S4: Summary of annualized change in eGFR; Table S5: Associations of eGFR definitions with comorbidities, stratified by sex.

## Abbreviations

The following abbreviations are used in this manuscript:

BMI	Body Mass Index
CKD	Chronic Kidney Disease
CKD-EPI	Chronic Kidney Disease Epidemiology Collaboration
DEXA	Dual-Energy X-ray Absorptiometry
eGFR	Estimated Glomerular Filtration Rate
eGFRLMISCr	Lean Mass Index-Adjusted Estimated Glomerular Filtration Rate
eGFRSCr	Standard Serum Creatinine-Based Estimated Glomerular Filtration Rate
FMI	Fat Mass Index
HbA1c	Glycated Hemoglobin
HDL-C	High-Density Lipoprotein Cholesterol
IDF	International Diabetes Federation
KDIGO	Kidney Disease: Improving Global Outcomes
LDEC	Lancet Diabetes and Endocrinology Commission
LEAD	Lung, hEart, social, body (name of the study cohort)
LMI	Lean Mass Index
LoA	Limits of Agreement
LDL-C	Low-Density Lipoprotein Cholesterol
OR	Odds Ratio
P30	Estimates Within 30% of the Reference Value
SCr	Serum Creatinine
WHO	World Health Organization
