# Supplementary material for: Lean Mass Index Adjustment of eGFR Strengthens Associations with Cardiometabolic Risk Factors
Source: Diagnostics (Basel). 2026 Oct 28;16(21):3540. doi: 10.3390/diagnostics16213540 (PMC13762420; doi:10.3390/diagnostics16213540)
Supplement: Supplementary file 1 [file diagnostics-16-03540-s001.zip › diagnostics-4596303-supplementary.pdf]

Supplementary Materials

Lean Mass Index Adjustment of eGFR Strengthens Associations with Cardiometabolic Risk Factors

Author list and Affiliations:

Oliver Helk[1,2], Charmaine J. M. Lim[3,4], Matthäus Metz[1], Emiel F. M. Wouters[3,4,5†], Robab Breyer-Kohansal[3,6†], Marie-Kathrin Breyer[3,7†]

[1]Department of Medicine III, Division for Nephrology and Dialysis, Medical University of Vienna, General Hospital of Vienna, Austria

[2]Austrian Academic Institute for Clinical Nutrition, Vienna, Austria

[3]Ludwig Boltzmann Institute for Lung Health, Vienna, Austria

[4]Sigmund Freud Private University, Faculty of Medicine, Vienna, Austria

[5]NUTRIM, Institute for Nutrition and Translational Research in Metabolism, Maastricht, Netherlands

[6] Department of Internal Medicine and Pulmonology, Clinic Hietzing, Vienna Healthcare Group, Vienna, Austria

[7] Department of Respiratory and Pulmonary Diseases, Site Penzing of Clinic Ottakring, Vienna Healthcare Group, Vienna, Austria.

[†]Authors contributed equally to this work as last authors.

Table S1. Baseline characteristics of included participants stratified by eGFR categories.

	Total	G1			G2			G3a			G3b			G4/5		
		$eGFR_{SCr}$	$eGFR_{LMISCr}$	p-value	$eGFR_{SCr}$	$eGFR_{LMISCr}$	p-value	$eGFR_{SCr}$	$eGFR_{LMISCr}$	p-value	$eGFR_{SCr}$	$eGFR_{LMISCr}$	p-value	$eGFR_{SCr}$	$eGFR_{LMISCr}$	p-value
Total N (%)	11141	6993 (62.8)	6910 (62.0)		3812 (34.2)	3810 (34.2)		264 (2.4)	353 (3.2)		62 (0.6)	61 (0.5)		10 (0.1)	7 (0.1)	
Males	5265 (47.3)	3338 (47.7)	3261 (47.2)	<0.001	1764 (46.3)	1806 (47.4)	<0.001	122 (46.2)	157 (44.5)	<0.001	35 (56.5)	37 (60.7)	<0.001	6 (60.0)	4 (57.1)	0.876
Age	46.0 (17.2)	38.1 (14.0)	38.3 (14.3)	0.404	58.3 (13.6)	57.3 (14.0)	0.001	69.7 (8.8)	68.3 (9.2)	0.028	71.7 (7.8)	70.9 (9.2)	0.625	73.8 (6.8)	72.1 (6.4)	0.717
18 - <25	1636 (14.7)	1557 (22.3)	1543 (22.3)	<0.001	78 (2.0)	92 (2.4)	<0.001	1 (0.4)	1 (0.3)	0.96	0 (0.0)	0 (0.0)	-	0 (0.0)	0 (0.0)	-
25 - <40	2880 (25.9)	2538 (36.3)	2485 (36.0)		340 (8.9)	392 (10.3)		2 (0.8)	2 (0.6)		0 (0.0)	1 (1.6)		0 (0.0)	0 (0.0)	
40 - <65	4604 (41.3)	2621 (37.5)	2548 (36.9)		1917 (50.3)	1945 (51.0)		55 (20.8)	101 (28.6)		10 (16.1)	9 (14.8)		1 (10.0)	1 (14.3)	
⩾65	2021 (18.1)	277 (4.0)	334 (4.8)		1477 (38.7)	1381 (36.2)		206 (78.0)	249 (70.5)		52 (83.9)	51 (83.6)		9 (90.0)	6 (85.7)	
Creatinine, mg/dL	0.8 (0.2)	0.8 (0.1)	0.8 (0.1)	0.027	0.9 (0.1)	0.9 (0.1)	<0.001	1.2 (0.2)	1.1 (0.2)	0.176	1.6 (0.3)	1.6 (0.3)	0.621	2.9 (2.0)	3.2 (2.3)	0.936
$Creatinine_{LMI}$, mg/dL	0.8 (0.2)	0.8 (0.2)	0.8 (0.1)	<0.001	0.9 (0.2)	0.9 (0.1)	<0.001	1.2 (0.2)	1.2 (0.2)	<0.001	1.5 (0.3)	1.6 (0.2)	0.287	2.7 (1.9)	3.2 (2.1)	0.725
$eGFR_{SCr}$	96.1 (19.1)	107.8 (12.0)	106.9 (13.4)	<0.001	78.7 (7.9)	81.2 (10.6)	<0.001	54.6 (4.0)	59.0 (8.3)	<0.001	39.7 (3.9)	40.1 (6.7)	0.686	22.8 (7.8)	21.7 (9.9)	0.681
$eGFR_{LMISCr}$	95.9 (20.0)	106.9 (14.1)	108.4 (12.3)	<0.001	79.5 (11.6)	78.0 (8.0)	<0.001	56.2 (7.4)	54.6 (3.9)	0.006	41.3 (6.5)	39.5 (3.9)	0.069	26.7 (11.0)	20.8 (8.5)	0.149
Height, cm	170.6 (9.5)	171.2 (9.5)	170.9 (9.5)	0.071	169.9 (9.4)	170.2 (9.5)	0.097	166.5 (9.0)	167.8 (9.5)	<0.001	167.9 (9.0)	169.3 (8.9)	0.434	168.0 (11.8)	166.3 (11.4)	0.369
Weight, kg	75.1 (16.0)	73.4 (16.1)	75.7 (16.9)	<0.001	77.6 (15.4)	73.9 (14.3)	<0.001	80.0 (14.8)	73.1 (14.2)	<0.001	82.8 (14.6)	81.2 (16.7)	0.544	90.0 (14.6)	77.3 (18.4)	0.05
Waist circumference, cm	93.0 (13.8)	90.4 (13.4)	92.2 (14.2)	<0.001	97.0 (13.0)	94.1 (12.8)	<0.001	102.7 (15.0)	96.6 (14.0)	<0.001	106.1 (12.5)	104.2 (14.3)	0.413	111.2 (12.4)	100.1 (13.7)	0.041
Waist-to-height, ratio	0.5 (0.1)	0.5 (0.1)	0.5 (0.1)	<0.001	0.6 (0.1)	0.6 (0.1)	<0.001	0.6 (0.1)	0.6 (0.1)	<0.001	0.6 (0.1)	0.6 (0.1)	0.221	0.7 (0.1)	0.6 (0.1)	0.163
LMI, kg/m^2	16.2 (2.3)	16.0 (2.3)	16.4 (2.4)	<0.001	16.5 (2.3)	15.8 (2.1)	<0.001	16.7 (2.0)	15.4 (2.0)	<0.001	17.2 (2.2)	16.8 (2.7)	0.385	18.8 (2.8)	16.6 (3.1)	0.031
ALMI, kg/m^2	7.4 (1.3)	7.4 (1.3)	7.6 (1.4)	<0.001	7.5 (1.3)	7.2 (1.2)	<0.001	7.5 (1.1)	6.9 (1.1)	<0.001	7.7 (1.2)	7.5 (1.4)	0.413	8.3 (1.7)	7.2 (1.8)	0.109
BMI, kg/m^2	25.7 (4.8)	25.0 (4.7)	25.8 (5.1)	<0.001	26.8 (4.6)	25.4 (4.2)	<0.001	28.8 (4.7)	25.9 (4.4)	<0.001	29.4 (4.8)	28.2 (4.7)	0.17	31.9 (4.5)	27.9 (5.8)	0.112
FMI, kg/m^2	8.7 (3.6)	8.2 (3.5)	8.6 (3.8)	<0.001	9.5 (3.6)	8.8 (3.2)	<0.001	11.3 (3.9)	9.7 (3.5)	<0.001	11.4 (3.9)	10.5 (3.1)	0.201	12.2 (3.7)	10.5 (3.7)	0.728
VATI, kg/m^2	0.3 (0.3)	0.2 (0.2)	0.3 (0.3)	<0.001	0.4 (0.3)	0.4 (0.3)	<0.001	0.6 (0.3)	0.5 (0.3)	0.003	0.7 (0.3)	0.6 (0.3)	0.408	0.8 (0.4)	0.6 (0.4)	0.145
Never smoker	5099 (45.8)	3195 (45.7)	3164 (45.8)	<0.001	1737 (45.6)	1724 (45.3)	0.695	135 (51.1)	182 (51.6)	0.47	28 (45.2)	26 (42.6)	0.638	4 (40.0)	3 (42.9)	0.277
Former smoker	3301 (29.7)	1734 (24.8)	1768 (25.6)		1426 (37.4)	1364 (35.8)		105 (39.8)	133 (37.7)		30 (48.4)	32 (52.5)		6 (60.0)	4 (57.1)	
Current smoker	2730 (24.5)	2056 (29.4)	1969 (28.5)		646 (17.0)	720 (18.9)		24 (9.1)	38 (10.8)		4 (6.5)	3 (4.9)		0 (0.0)	0 (0.0)	
Packyears	9.3 (17.8)	7.7 (15.3)	7.9 (15.5)	0.783	11.8 (20.5)	11.4 (20.2)	0.472	14.2 (27.0)	13.5 (25.4)	0.865	19.4 (27.1)	18.0 (27.4)	0.797	15.1 (30.9)	20.6 (36.4)	0.978
Metabolic syndrome	2947 (26.5)	1215 (17.4)	1420 (20.5)	<0.001	1506 (39.5)	1296 (34.0)	<0.001	172 (65.2)	183 (51.8)	1	45 (72.6)	42 (68.9)	0.114	9 (90.0)	6 (85.7)	1
$Obesity_{WHO}$	1888 (16.9)	955 (13.7)	1303 (18.9)	<0.001	802 (21.0)	500 (13.1)	0.18	97 (36.7)	59 (16.7)	0.104	26 (41.9)	22 (36.1)	0.947	8 (80.0)	4 (57.1)	0.876
$Obesity_{LDEC}$	4823 (43.3)	2367 (33.8)	2731 (39.5)	<0.001	2202 (57.8)	1846 (48.5)	0.001	195 (73.9)	196 (55.5)	0.816	50 (80.6)	46 (75.4)	1	9 (90.0)	4 (57.1)	-
Dyslipidemia	8168 (84.2)	4494 (79.0)	4491 (79.8)	<0.001	3369 (91.7)	3291 (90.1)	0.959	237 (90.8)	322 (92.5)	0.616	58 (93.5)	57 (93.4)	0.619	10 (100.0)	7 (100.0)	-

Pre-diabetes/diabetes	2900 (26.1)	1303 (18.7)	1394 (20.3)	0.022	1392 (36.7)	1294 (34.2)	<0.02	159 (60.7)	173 (49.1)	0.005	38 (62.3)	35 (58.3)	0.712	8 (80)	4 (57.1)	0.593
Pre-diabetes	2304 (20.8)	1056 (15.2)	1103 (16.0)	0.16	1111 (29.3)	1046 (27.6)	0.103	110 (42.0)	131 (37.2)	0.243	20 (32.8)	20 (33.3)	1	7 (70)	4 (57.1)	0.644
Diabetes	596 (5.3)	247 (3.5)	291 (4.2)	0.039	281 (7.4)	248 (6.5)	0.149	49 (18.6)	42 (11.9)	0.022	18 (29.0)	15 (24.6)	0.684	1 (10.0)	0 (0.0)	-
Cardiovascular disease	804 (7.2)	197 (2.8)	229 (3.3)	0.105	494 (13.0)	458 (12.0)	0.229	76 (28.8)	78 (22.1)	0.316	31 (50.0)	35 (57.4)	0.712	6 (60.0)	4 (57.1)	0.546
Hypertension	1890 (17.0)	553 (7.9)	675 (9.8)	<0.001	1128 (29.6)	1010 (26.5)	0.556	152 (57.6)	154 (43.6)	0.718	48 (77.4)	46 (75.4)	0.968	9 (90.0)	5 (71.4)	0.257
Osteopenia/osteoporosis	5071 (46.6)	2790 (40.9)	2611 (38.8)	0.159	2061 (55.3)	2164 (58.0)	<0.001	174 (67.7)	249 (71.8)	0.248	41 (67.2)	41 (69.5)	0.715	5 (55.6)	6 (85.7)	1
Asthma	1033 (9.3)	682 (9.8)	673 (9.8)	0.26	327 (8.6)	325 (8.6)	0.312	17 (6.5)	28 (8.1)	0.936	7 (11.3)	6 (9.8)	0.274	0 (0.0)	1 (14.3)	-
COPD	839 (7.6)	455 (6.5)	435 (6.3)	0.005	340 (8.9)	341 (9.0)	0.105	38 (14.4)	55 (15.6)	1	6 (9.7)	8 (13.1)	0.001	0 (0.0)	0 (0.0)	-
Anxiety	2311 (20.9)	1578 (22.8)	1515 (22.1)	0.104	685 (18.0)	731 (19.2)	0.02	38 (14.4)	52 (14.8)	1	8 (12.9)	11 (18.0)	0.297	2 (20.0)	2 (28.6)	0.895
Depression	1044 (9.4)	651 (9.4)	626 (9.1)	0.211	339 (8.9)	361 (9.5)	0.416	39 (14.9)	41 (11.7)	1	12 (19.7)	13 (21.3)	0.055	3 (30.0)	3 (42.9)	0.876
Psychological disorders	715 (6.4)	412 (5.9)	421 (6.1)	0.037	276 (7.2)	261 (6.9)	0.524	21 (8.0)	24 (6.8)	0.477	4 (6.5)	8 (13.1)	0.135	2 (20.0)	1 (14.3)	1
Neurological disorders	357 (3.2)	168 (2.4)	187 (2.7)	0.057	160 (4.2)	139 (3.6)	0.251	23 (8.7)	25 (7.1)	1	5 (8.1)	5 (8.2)	1	1 (10.0)	1 (14.3)	1
Systolic blood pressure, mmHg	129.0 (20.8)	124.0 (18.7)	124.8 (19.1)	0.043	136.9 (21.4)	135.3 (21.7)	0.001	140.0 (20.3)	138.3 (20.7)	0.803	148.3 (22.2)	148.5 (22.5)	0.956	150.2 (13.4)	138.3 (21.8)	0.13
Diastolic blood pressure, mmHg	77.1 (11.4)	75.5 (11.2)	75.9 (11.3)	0.068	80.0 (11.3)	79.2 (11.3)	0.002	79.0 (11.1)	78.6 (11.0)	0.666	81.6 (12.1)	80.2 (12.6)	0.529	72.2 (10.4)	71.4 (10.4)	0.905
Pulse wave velocity, m/s	7.2 (2.3)	6.6 (1.8)	6.6 (1.9)	0.075	8.1 (2.6)	8.0 (2.6)	0.004	9.5 (3.2)	9.4 (3.1)	0.256	9.7 (2.4)	9.6 (2.4)	0.681	11.8 (3.7)	11.6 (4.6)	0.806
Cholesterol, mg/dL	203.6 (43.3)	195.7 (41.8)	195.4 (41.7)	0.545	218.1 (42.3)	217.4 (42.0)	0.451	205.5 (42.8)	217.2 (46.6)	0.006	198.0 (48.8)	202.7 (49.2)	0.537	184.3 (42.9)	190.7 (42.1)	0.397
HDL, mg/dL	64.0 (18.7)	63.1 (18.0)	62.1 (17.9)	0.003	65.9 (19.7)	67.3 (19.5)	0.003	61.2 (17.3)	65.4 (18.8)	0.23	56.2 (18.9)	58.2 (19.8)	0.471	50.0 (21.8)	48.6 (19.6)	0.918
LDL, mg/dL	123.1 (37.9)	118.4 (37.1)	118.4 (37.2)	0.986	130.4 (37.8)	129.3 (37.5)	0.251	117.6 (37.8)	127.2 (42.0)	0.004	109.9 (47.9)	114.7 (45.2)	0.583	98.8 (35.6)	105.4 (42.4)	0.336
Fibrinogen, g/L	3.1 (0.7)	3.0 (0.7)	3.0 (0.7)	0.202	3.3 (0.7)	3.2 (0.7)	0.012	3.5 (0.8)	3.5 (0.7)	0.882	3.7 (0.7)	3.7 (0.8)	0.937	4.3 (0.8)	4.3 (0.6)	0.927
Triglyceride, mg/dL	109.7 (72.3)	103.4 (72.3)	105.9 (72.2)	0.045	118.0 (70.1)	113.9 (72.0)	0.011	135.9 (65.7)	127.9 (59.0)	0.435	168.3 (94.2)	153.7 (88.1)	0.363	207.0 (158.1)	254.4 (186.0)	0.79
HbA1C, mmol/mol	34.3 (5.8)	33.4 (5.7)	33.6 (6.0)	0.011	35.5 (5.4)	35.1 (5.3)	0.002	38.9 (7.1)	37.2 (5.8)	0.002	39.8 (7.5)	38.9 (7.6)	0.503	38.6 (9.8)	34.7 (5.7)	0.573

Data are presented as mean (standard deviation) or N (%). P-values compare $eGFR_{SCr}$ and $eGFR_{LMISCr}$ within each chronic kidney disease stage. eGFR strata were defined as G1, G2, G3a, G3b, and G4/5. eGFR, estimated glomerular filtration rate; LMI, lean mass index; SCr, serum creatinine; COPD, chronic obstructive pulmonary disease; HDL, high-density lipoprotein; LDL, low-density lipoprotein; and HbA1C, glycated haemoglobin. Statistical significance was defined as p<0.05.

Table S2. Reclassification between raw and LMI-adjusted CKD-EPI 2009 eGFR categories according to LMI percentile range.

		LMI percentile range					
**$eGFR_{SCr}$**	**$eGFR_{LMISCr}$**	**<P10 (<13.27 kg/m²)**	**P10-P25 (13.28 - 14.35 kg/m²)**	**P25-P50 (14.35 - 16.02 kg/m²)**	**P50-P75 (16.02 - 17.83 kg/m²)**	**P75-P90 (17.83 - 19.26 kg/m²)**	**≥P90 (≥19.26 kg/m²)**
⩾90	⩾90	629 (56.5%)	953 (57.0%)	1555 (55.8%)	1502 (53.9%)	960 (57.5%)	621 (55.7%)
⩾90	60-<90	230 (20.6%)	176 (10.5%)	184 (6.6%)	176 (6.3%)	6 (0.4%)	0 (0.0%)
⩾90	45-<60	1 (0.1%)	0 (0.0%)	0 (0.0%)	0 (0.0%)	0 (0.0%)	0 (0.0%)
60-<90	⩾90	0 (0.0%)	1 (0.1%)	94 (3.4%)	190 (6.8%)	156 (9.3%)	249 (22.3%)
60-<90	60-<90	197 (17.7%)	464 (27.8%)	837 (30.1%)	788 (28.3%)	479 (28.7%)	201 (18.0%)
60-<90	45-<60	47 (4.2%)	48 (2.9%)	30 (1.1%)	30 (1.1%)	1 (0.1%)	0 (0.0%)
45-<60	60-<90	0 (0.0%)	0 (0.0%)	9 (0.3%)	27 (1.0%)	17 (1.0%)	18 (1.6%)
45-<60	45-<60	2 (0.2%)	23 (1.4%)	59 (2.1%)	56 (2.0%)	35 (2.1%)	8 (0.7%)
45-<60	30-<45	5 (0.4%)	2 (0.1%)	2 (0.1%)	1 (0.0%)	0 (0.0%)	0 (0.0%)
30-<45	60-<90	0 (0.0%)	0 (0.0%)	0 (0.0%)	0 (0.0%)	0 (0.0%)	1 (0.1%)
30-<45	45-<60	0 (0.0%)	0 (0.0%)	2 (0.1%)	6 (0.2%)	1 (0.1%)	4 (0.4%)
30-<45	30-<45	1 (0.1%)	4 (0.2%)	12 (0.4%)	8 (0.3%)	12 (0.7%)	9 (0.8%)
30-<45	15-<30	1 (0.1%)	0 (0.0%)	1 (0.0%)	0 (0.0%)	0 (0.0%)	0 (0.0%)
15-<30	30-<45	0 (0.0%)	0 (0.0%)	0 (0.0%)	1 (0.0%)	1 (0.1%)	3 (0.3%)
15-<30	15-<30	0 (0.0%)	0 (0.0%)	0 (0.0%)	0 (0.0%)	1 (0.1%)	1 (0.1%)
15-<30	<15	1 (0.1%)	0 (0.0%)	0 (0.0%)	0 (0.0%)	0 (0.0%)	0 (0.0%)
<15	15-<30	0 (0.0%)	0 (0.0%)	0 (0.0%)	0 (0.0%)	1 (0.1%)	0 (0.0%)
<15	<15	0 (0.0%)	0 (0.0%)	0 (0.0%)	0 (0.0%)	1 (0.1%)	0 (0.0%)

Data are presented as n (%). Percentages represent the proportion of participants within each LMI percentile range. Rows indicate CKD-EPI 2009 eGFR categories based on serum creatinine without LMI adjustment ($eGFR_{SCr}$), and columns indicate the corresponding categories after adjustment of serum creatinine for lean mass index (LMI; $eGFR_{LMISCr}$). LMI percentile ranges were defined according to the distribution of LMI in the study population. CKD-EPI 2009 eGFR categories were defined as <15, 15–<30, 30–<45, 45–<60, 60–<90, and ≥90 mL/min/1.73 m². Reclassification is represented by movement from the $eGFR_{SCr}$ to the $eGFR_{LMISCr}$.

Table S3. Overall reclassification between raw and LMI-adjusted CKD-EPI 2009 eGFR categories across LMI percentile ranges.

**LMI percentile range**	**LMI range**	**Observations, n/N (%)**
<P10	<13.27 kg/m^2	285/1114 (25.6)
P10-P25	13.28 - 14.35 kg/m^2	227/1671 (13.6)
P25-P50	14.35 - 16.02 kg/m^2	322/2785 (11.6)
P50-P75	16.02 - 17.83 kg/m^2	431/2785 (15.5)
P75-P90	17.83 - 19.26 kg/m^2	183/1671 (11.0)
≥P90	≥19.26 kg/m^2	275/1115 (24.7)

Data are presented as n and percentage of participants within each LMI percentile range. Reclassification was defined as a change in CKD-EPI 2009 eGFR category between the unadjusted estimate based on measured serum creatinine ($eGFR_{SCr}$) and the estimate obtained after adjustment of serum creatinine for lean mass index (LMI; $eGFR_{LMISCr}$). LMI percentile ranges were defined according to the distribution of LMI in the study population. CKD-EPI 2009 eGFR categories were defined as <15, 15–<30, 30–<45, 45–<60, 60–<90, and ≥90 mL/min/1.73 m^2.

Table S4. Summary of annualized change in eGFR.

Metric	eGFRSCr	eGFRLMISCr
Mean annual slope	-0.41 ± 2.28	-0.75 ± 2.08
Mean paired difference	-0.34 ± 3.00	
p-value	<0.001	

Table S5. Associations of eGFR definitions with comorbidities, stratified by sex.

	$eGFR_{SCr}$				$eGFR_{LMISCr}$			
	**Male**		**Female**		**Male**		**Female**	
**Outcomes**	**OR (95% CI)**	**p-value**	**OR (95% CI)**	**p-value**	**OR (95% CI)**	**p-value**	**OR (95% CI)**	**p-value**
Cardiovascular disease	0.985 (0.977, 0.993)	<0.001	0.999 (0.99, 1.008)	0.836	0.995 (0.988, 1.003)	0.233	1.011 (1.002, 1.02)	0.018
Hypertension	0.983 (0.976, 0.989)	<0.001	0.997 (0.99, 1.003)	0.303	1.002 (0.996, 1.008)	0.474	1.022 (1.016, 1.029)	<0.001
Metabolic syndrome	0.995 (0.990, 1.000)	0.050	1.001 (0.995, 1.006)	0.823	1.023 (1.018, 1.028)	<0.001	1.032 (1.026, 1.037)	<0.001
Dyslipidemia	0.995 (0.989, 1.002)	0.201	1.004 (0.998, 1.01)	0.205	1.006 (1.000, 1.012)	0.066	1.011 (1.006, 1.016)	<0.001
Prediabetes	1.004 (0.998, 1.010)	0.161	1.002 (0.996, 1.008)	0.529	1.011 (1.005, 1.016)	<0.001	1.018 (1.012, 1.023)	<0.001
Diabetes	1.012 (1.003, 1.022)	0.013	1.007 (0.995, 1.019)	0.243	1.036 (1.026, 1.046)	<0.001	1.049 (1.036, 1.062)	<0.001
$Obesity_{LDEC}$	0.995 (0.990, 1.000)	0.061	1.008 (1.003, 1.012)	0.001	1.042 (1.037, 1.047)	<0.001	1.054 (1.049, 1.059)	<0.001
$Obesity_{WHO}$	0.996 (0.991, 1.002)	0.203	1.001 (0.995, 1.007)	0.824	1.064 (1.057, 1.072)	<0.001	1.088 (1.080, 1.097)	<0.001

Estimates are odds ratios (OR) and 95% confidence intervals (CI) obtained from logistic regression models adjusted for age. Statistical significance was defined as p<0.05.

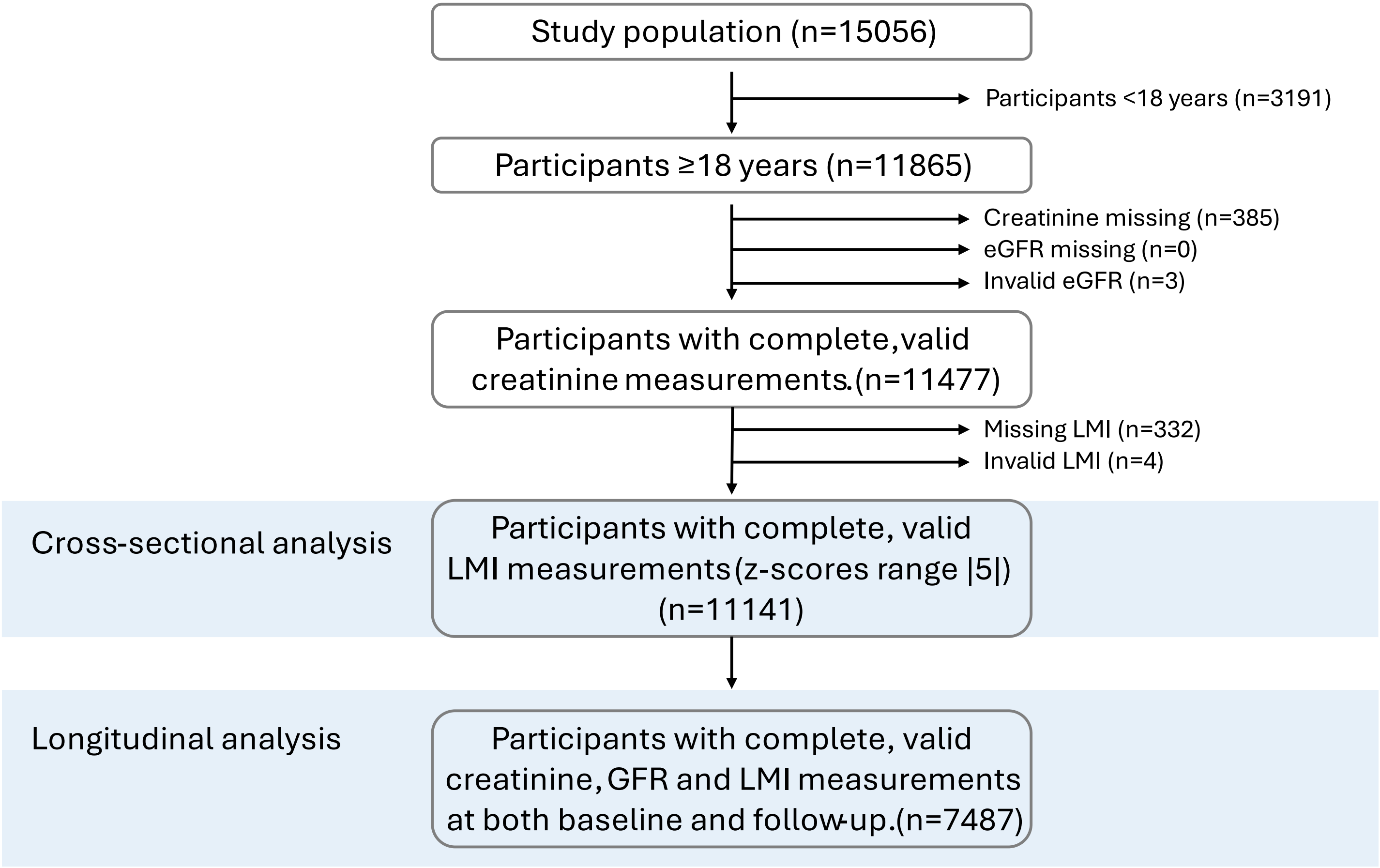

Figure S1. Study population flow chart. Data originate from the Austrian LEAD cohort (18).

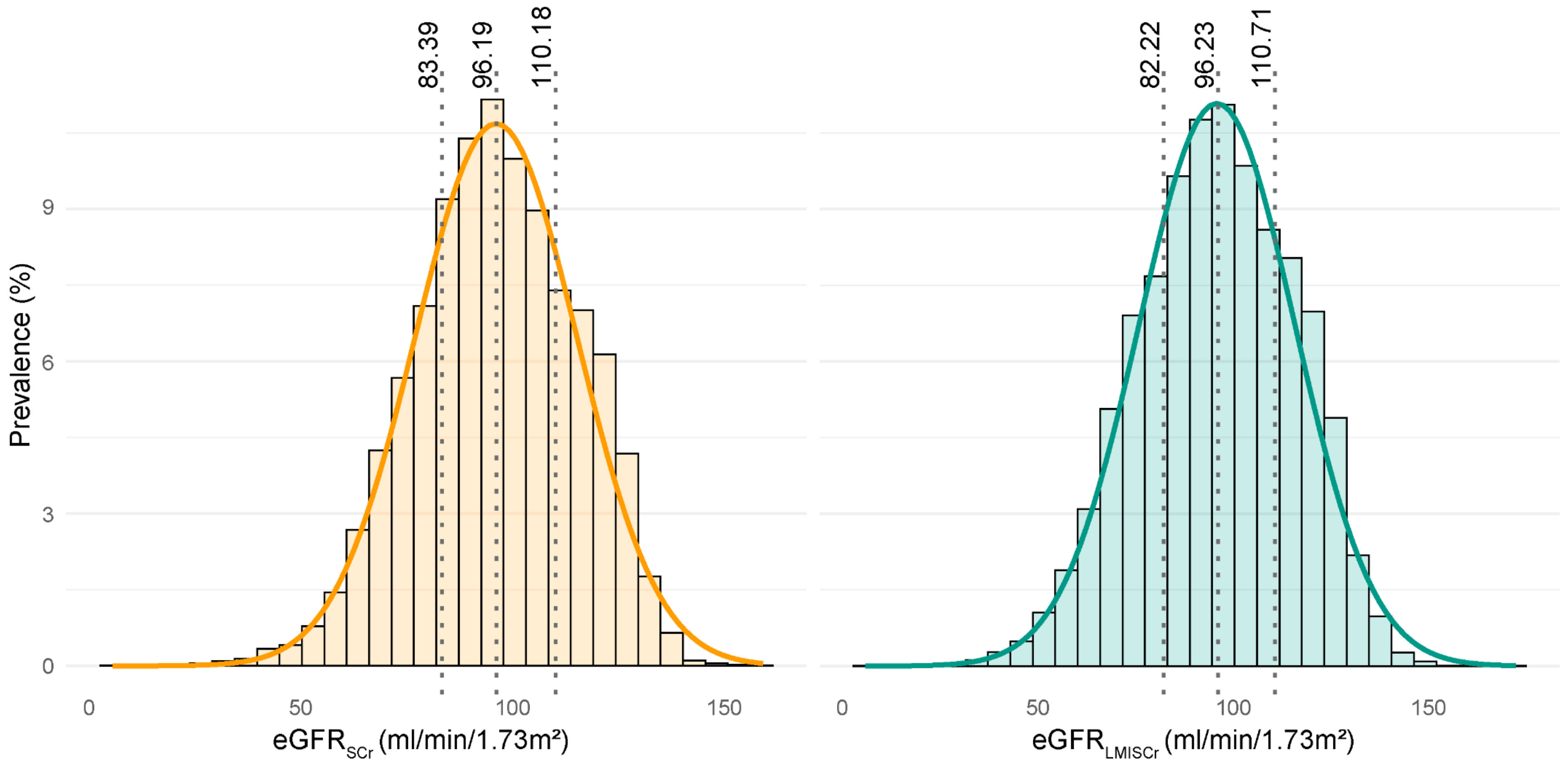

Figure S2. Distribution of eGFR in the total population. Legend: The distributions are fitted with Gaussian curves, and the labels indicate the 25th, 50th and 95th percentiles.

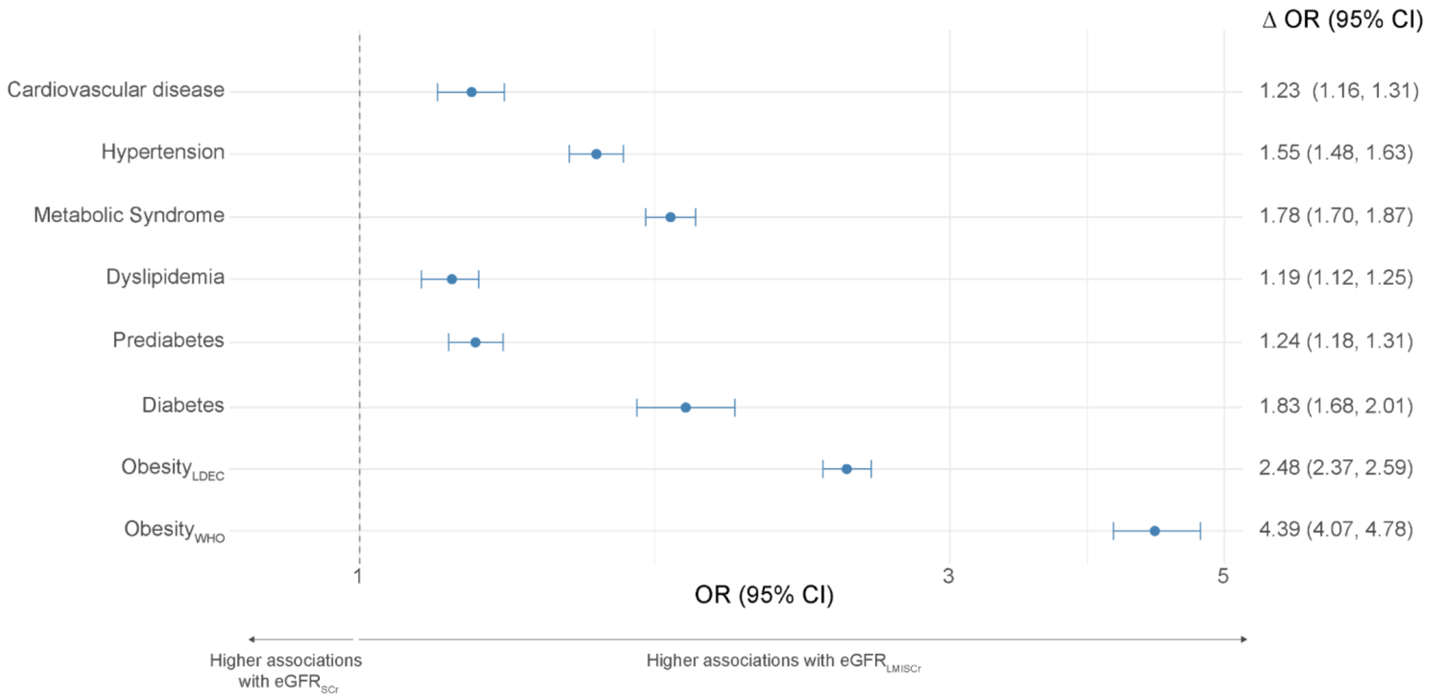

Figure S3. Comparisons of outcome associations between eGFR definitions. *Figure legend: Points and error bars show ratios of odds ratios (ΔOR) and their 95% confidence intervals from age- and sex-adjusted logistic regression models. ΔOR is the OR obtained using* $eGFR_{LMISCr}$ *divided by the OR obtained using* $eGFR_{SCr}$*. Both eGFR variables were standardized separately, and ORs are expressed per 1-SD higher eGFR. Confidence intervals were obtained from 1,000 paired participant-level bootstrap resamples. The null ratio is 1; ratios >1 or <1 indicate numerically higher or lower ORs, respectively, for* $eGFR_{LMISCr}$*. They do not, by themselves, indicate stronger or weaker associations; interpretation requires the individual ORs in Table 2.*

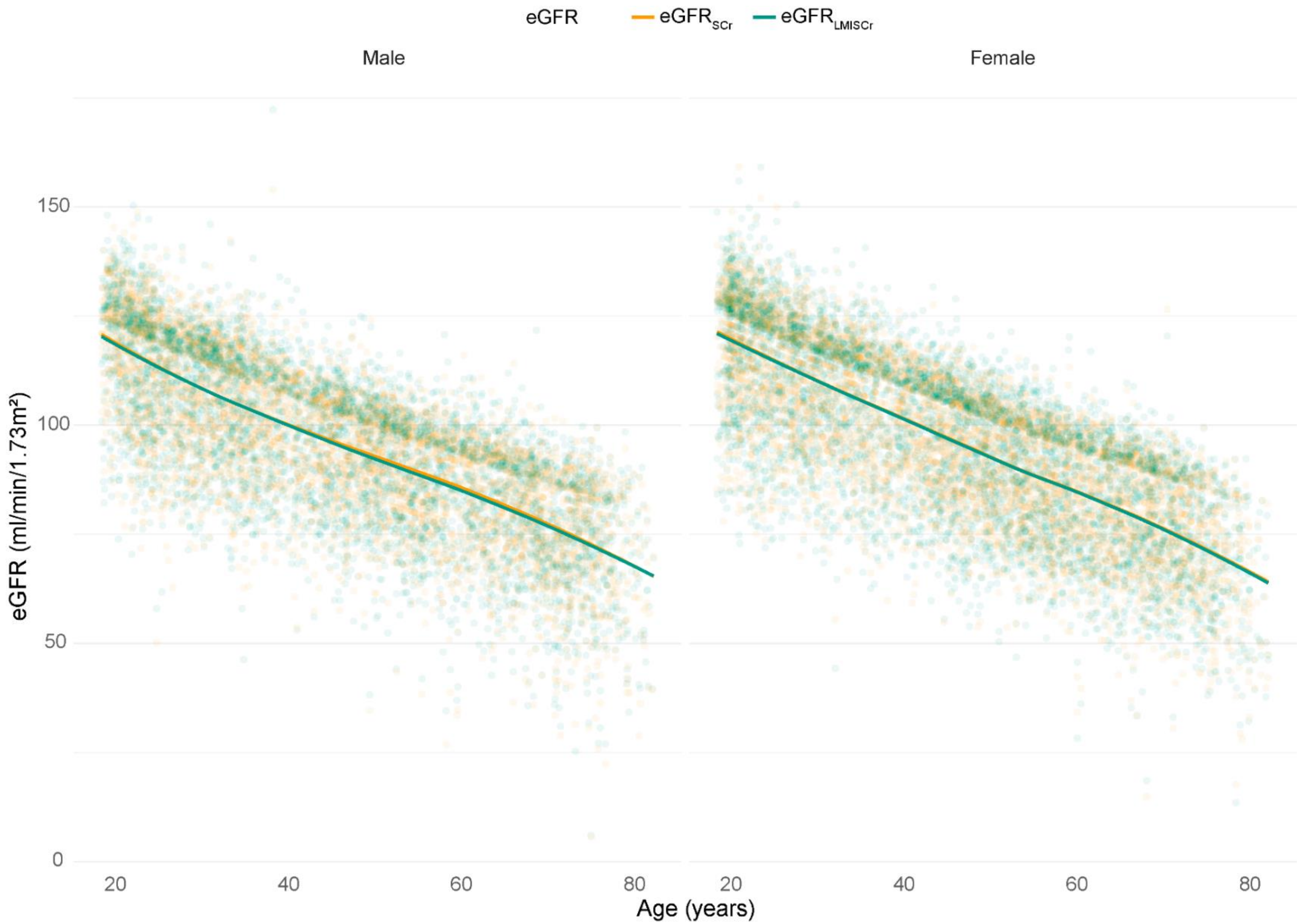

Figure S4. Distribution of eGFR by age and sex.

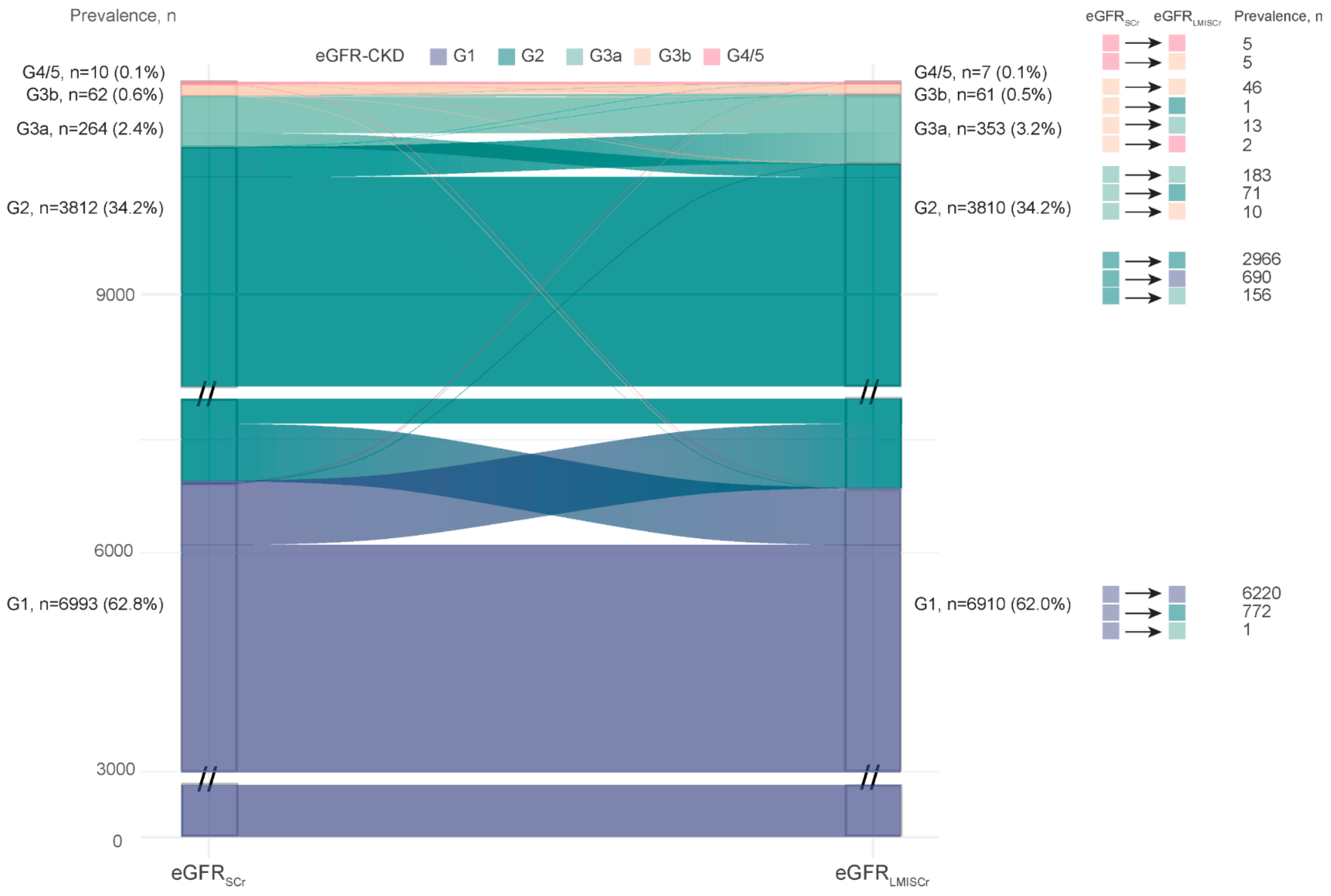

Figure S5. Transitions between GFR categories by eGFR definition. *Figure legend: flows between boxes represent reclassification according to the alternative eGFR equation and do not establish whether the resulting classification represents a correct or incorrect diagnosis, as no independent clinical reference standard was available.*

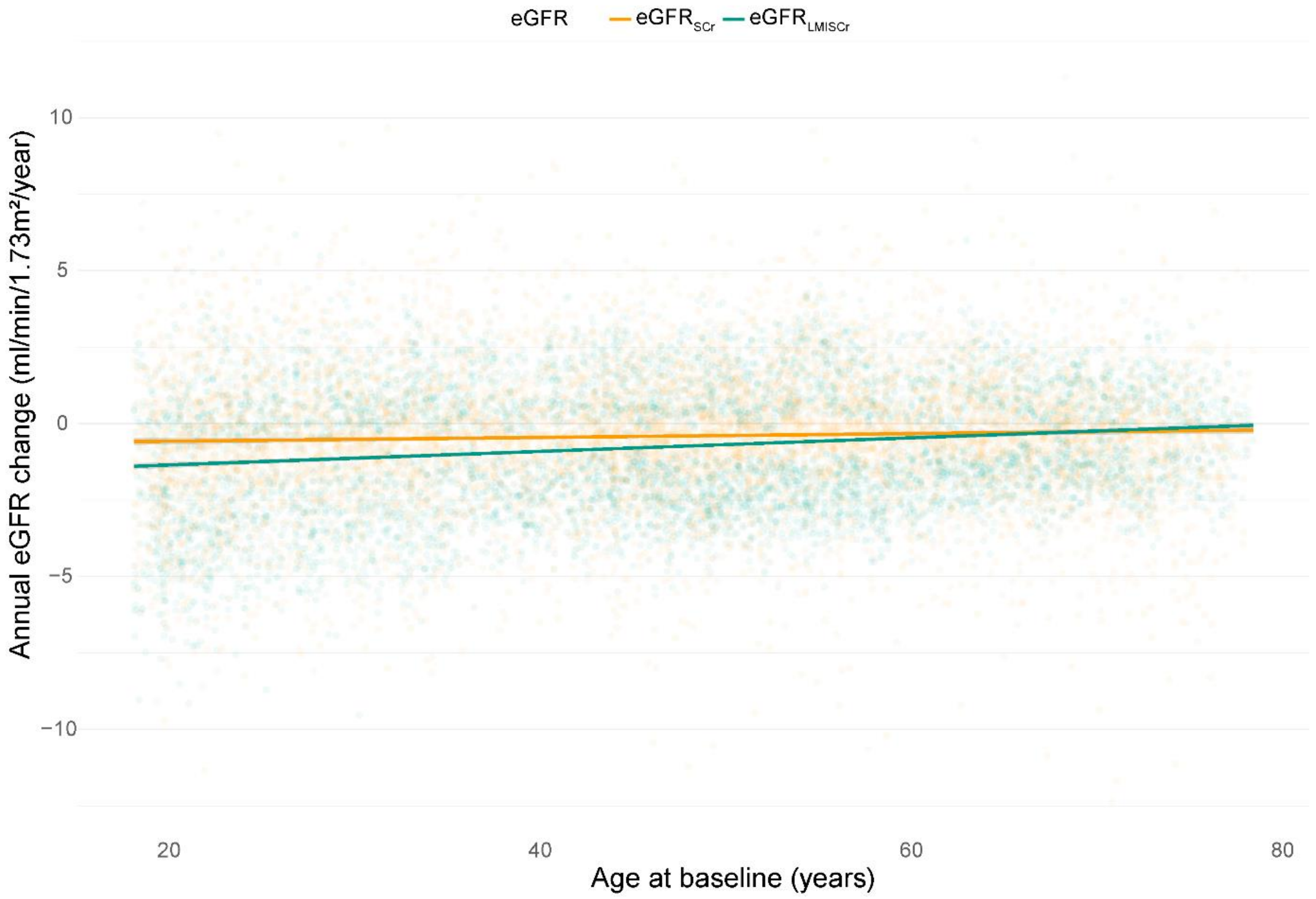

Figure S6. Annualized change in eGFR.
